# Investigation of a Three-Dimensional Micro-Scale Sensing System Based on a Tapered Self-Assembly Four-Cores Fiber Bragg Grating Probe

**DOI:** 10.3390/s18092824

**Published:** 2018-08-27

**Authors:** Kunpeng Feng, Jiwen Cui, Xun Sun, Hong Dang, Tangjun Shi, Yizhao Niu, Yihua Jin, Jiubin Tan

**Affiliations:** Institute of Ultra-precision Optoelectronic Instrument Engineering, Harbin Institute of Technology, Science Park, No.2 Yikuang Street, Nangang District, Harbin 150080, China; sx2503962673@163.com (X.S.); sired_hit@163.com (H.D.); 13115312773@163.com (T.S.); m18846819526@163.com (Y.N.); jin_yihua@foxmail.com (Y.J.); jbtan@hit.edu.cn (J.T.)

**Keywords:** micro-scale sensing, FBG, tapered four-cores fiber, self-assembly

## Abstract

Three-dimensional micro-scale sensors are in high demand in the fields of metrology, precision manufacturing and industry inspection. To extend the minimum measurable dimension and enhance the accuracy, a tapered four-cores fiber Bragg grating (FBG) probe is proposed. The sensing model is built to investigate the micro-scale sensing characteristics of this method and the design of the tapered stylus is found to influence the accuracy. Therefore, a π/2 phase-shift point is introduced into the FBGs comprised in the probe to suppress spectrum distortion and improve accuracy. Then, the manufacturing method based on capillary self-assembly is proposed to form the probe and the critical length to form a square array for four cylindrical fibers is verified to be effective for the tapered fibers. Experimental results indicate that the design of the tapered stylus can extend the minimum measurable dimension by twofold and has nearly no influence on its sensitivity. The three-dimensional measurement repeatability is better than 31.1 nm and the stability is better than 200 nm within once measuring process. Furthermore, the measurement precision of the three-dimensional micro-scale measurement results is less than 150 nm. It would be widely used in measuring micro-scale features for industry inspection or metrology.

## 1. Introduction

The design and manufacture of three-dimensional microstructures on core components has become an upward trend in high-end equipment manufacturing industries [1]. For example, there is a large number of cooling cavity structures on the complex surface of a turbine blade in aero-engines [2,3]. These cavity structures have several three-dimensional structural characteristics, such as forward-expanded type and crescent type [4]. Meanwhile, their diameters are in a range from 300 to 500 μm. In the fuel injection system of aero-engines, injection holes are located on the concentric injection loops of different angles, and their diameter and depth can reach 200 μm and 2 mm, respectively [1,3]. Besides, the thrust performance directly relates to the accuracy of manufacture. Research indicates that the maximum thrust could decrease by ~17% when the manufacture errors are over 10% [5]. The first is that the key to accomplish precise manufacture of three-dimensional micro structures begins with solving the three-dimensional microscale sensing problems [1]. Therefore, a direct and effective method is in high demand to evaluate the three-dimensional manufacturing results of microstructures.

Many works have been published on in this particular aspect in recent years and several tactile probing systems have been proposed. The existing probing methods can be generally classified into three types: flexure mechanics (hinge or cantilever) probes [6], vibration probes [7], and optical fiber probes [8,9,10]. In a flexure mechanics probing system, there is a contradiction between the stiffness of the stylus and the flexibility of the hinge or cantilever [6]. Due to the design based on the stability of the probing system, the ratio of the measurable depth to the minimum measurable dimension can’t satisfy the requirements of current three-dimensional microscale measurements. Though the stylus of a vibration probing system can be miniaturized to several microns and the probing force can also be decreased to sub mN values, one can only determine only one or two measurable dimensions [7]. The measurement procedure is very complex and it is more suitable for surface roughness measurements but not position or geometry. The principles of the optical fiber probing system are not unified. The probing system from PTB (Physikalisch-Technische Bundesanstalt) based on microball scattering can achieve three-dimensional measurements but its measurable depth is limited by the block of the side walls of microparts [8]. The probing system based on orthogonal imaging of the fiber stylus has a bulky sensing system and has risk of mechanical interference with the measured parts [9]. On the other hand, its axial sensing mechanism employing a buckling stylus is not stable. Conventional probing methods could not satisfy the microscale measurement requirements of the high-end equipment manufacturing industries because of the minimum measurable dimensions, the maximum measurable depth and the three-dimensional sensing capacity. Four-cores FBG (fiber Bragg grating) probing systems have solved several problems of microscale measurement [10]. However, their structure is restricted by the availability of commercial four-core fibers, and the signal transmission loss and cost is very high.

Tapered FBG sensors could reduce the size of optical fiber sensors and improve their performance. Xu reported a chirped FBG sensor in a tapered optical fiber [11]. The taper structure induces a strain gradient along the FBG. The effective bandwidth is employed to measure the strain which shows excellent temperature-independent behavior. A tapered FBG for simultaneously measuring strain and temperature was proposed by Lima and the uncoupling relationship between strain and the full width at half maximum was found in a linear chirp FBG [12]. To solve the axial/transverse forces crosstalk in microsurgical instruments, Abushagur built a tapered FBG model to investigate the different responses of FBG and tapered FBG to axial/transverse forces [13]. In 2006, Osuch proposed a novel tailored intrinsic chirped and tapered FBG sensor [14]. This sensor, which included chirp enhancement and chirp reduction structures could significantly increase the sensitivity and reduce the influence of F-P resonance formation results from higher elongation of the thinner end of the tapered FBG. After that, Osuch first designed a chirped tapered FBG in double-pass configuration [15]. This novel double-pass structure has two reflection bands, of which only one is sensitive to bending, so the tilt angle and temperature measurement can be simultaneously achieved. In 2017, Konrad and Osuch reported a linearly chirped tapered FBG based Fabry–Perot cavity sensor [16]. The standard deviation of the derivative of the reflection spectrum is first utilized to sense applied strain and temperature is measured by monitoring wavelength shift. The development of tapered FBG sensors improved the related theory. However, it is very difficult to accomplish wiring an intrinsic chirp tapered FBG. On the other hand, signal demodulation through an optical spectrum analyzer (OSA) cannot be real-time and the complex spectrum of a linearly chirped and tapered FBG has a significant influence on sensing accuracy. Furthermore, how to deal with the nonlinearly chirped tapered FBG remains an open issue.

Based on the investigation of the conventional four-cores FBG probes [10] and tapered FBG sensors [11,12,13,14,15,16], a tapered self-assembly four-cores FBG probe is proposed in this paper. This probe is manufactured using four tapered single-core FBGs through the self-assembly technique, so there is no multicore fiber fanouts in the probing system, and the signal transmission loss and cost can be significantly decreased. On the other hand, the quality of the spectral signals is also improved. Its structure design is more flexible and the diameter of the tapered stylus could be smaller. The maximum taper ratio of the proposed probe can reach higher than 2:1, the spherical tip is smaller than 100 μm, the three-dimensional tactile resolution is higher than 30 nm and the repeatability is higher than 31.1 nm.

This paper is organized as follows: Section 2 demonstrates the sensing principle of the tapered self-assembly four-cores FBG probe and establishes the relationship between its structural design and sensing performance. Section 3 describes the self-assembly manufacture process and experiments on sensing characteristics testing. Finally, conclusions are presented.

## 2. Sensing Principle

**Figure 1:**
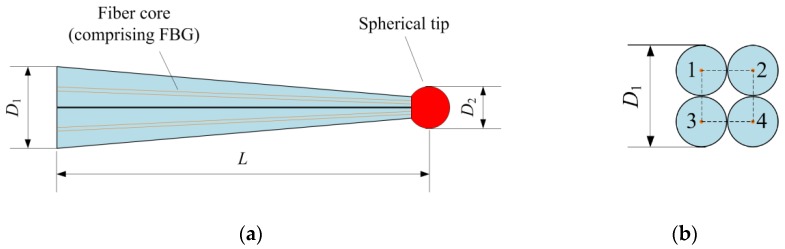
The schematic diagram of the proposed tapered four-cores FBG probe: (**a**) Parallel corss-section view; (**b**) Perpendicular corss-section view.

The schematic diagram of the proposed tapered four-cores FBG probe is shown in Figure 1. This probe consists of a tapered stylus formed by four FBGs and a spherical tip. These four fibers are arrayed in a square array. The sensing principle of the tapered four-cores FBG probe is based on the inner FBGs subjected to the distributed strain induced by the deformation of the stylus.

During radial and axial tactile sensing, the tapered four-cores FBG probe can be simplified into the cantilever and compressed beam model, so the relationship between the reflection spectra of the FBGs and the tactile displacements can be established. In this section, the sensing principle and sensing characteristics of the tapered four-cores FBG probe are demonstrated. First, radial and axial sensing models are built to demonstrate the tactile sensing mechanism of the tapered four-cores FBG probe. Then, a numerical solution for solving the nonlinear chirp FBG spectrum is utilized to achieve the relationship between the probe signal and the tactile displacements. The taper is found to have significant influences on the spectrum distortion, and the fine spectrum induced by a π/2 phase-shift point is verified to have effective improvement on the sensing accuracy. Finally, the sensing characteristics of the tapered four-cores phase-shift FBG probe are investigated. The tapered four-cores phase-shift FBG probe could improve the sensitivity when the diameter of the stylus is decreased. On the other hand, there is a decoupling signal processing method when the four cores are in a square array.

### 2.1. Sensing Principle of the Radial Displacement

**Figure 2:**
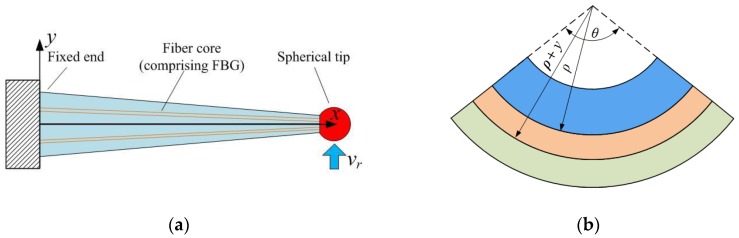
The analysis model for the radial tactile sensing: (**a**) Cantilever model; (**b**) Schematic diagram of deflected element, ρ is the curvature radius, y is the distance to neutral plane and θ is the radian of the deflected element.

The tapered four-cores FBG probe is deflected under a radial tactile displacement. To establish its radial sensing mechanism, it can be simplified into a cantilever model in Figure 2a. As it is shown in Figure 2b, there is a neutral plane in a deflected element. Based on the analysis of the deflected element, the differential equation of the deflection curve can be expressed as:

(1) $$\frac{d^{2}v_{r}}{dx^{2}}=-\frac{F_{r}\left(L-x\right)}{EI(x)}$$

wherein, $v_{r}(x)$ is the deflection curve, $F_{r}$ is the radial tactile force, L is the length of the stylus, E is the Young's modulus of the probe, x is the coordinate of the element, I(x) is the moment of inertia of the tapered stylus:

(2) $$I(x)=\frac{\pi }{64}{\left(D_{1}-\frac{D_{1}-D_{2}}{L}x\right)}^{4}$$

wherein, $D_{1}$ is the diameter of the fixed end, $D_{2}$ is the diameter of the free end, their ratio can be defined as $k=D_{1}/D_{2}$.

At the fixed end, the displacement and curvature of the tapered stylus is 0. These two boundary conditions can be expressed as:

(3) $$\left\{ \begin{array}{l} v_{r}(0)=0\\ v_{r}{}^{\prime }(0)=\theta (0)=0 \end{array}\right.$$

Substituting Equation (3) into Equation (1), the solution of the differential equation can be written as:

(4) $$v_{r}(x)=\frac{F_{r}L^{2}x^{2}\left(2x+3kL-3kx\right)}{6EI_{2}k^{3}{\left(x+kL-kx\right)}^{2}}$$

wherein, $I_{2}$ is the moment of inertia of the free end.

The relationship between the radial displacement and tactile force can be achieved when x=L:

(5) $$v_{r}=v_{r}(L)=\frac{F_{r}L^{3}}{3EI_{2}k^{3}}$$

Basing on Equations (4) and (5), the distributed stress within the tapered stylus can be written as:

(6) $$\sigma_{x}(x,y)=-E\frac{y}{\rho (x)}=-E\frac{d^{2}v_{r}}{dx^{2}}y=-\frac{3Ek^{3}\left(L-x\right)y}{L^{3}{\left(k-\frac{k-1}{L}x\right)}^{4}}v_{r}$$

wherein, y is the distance between the deflected element and neutral plane, ρ(x) is the curvature function.

**Figure 3:**
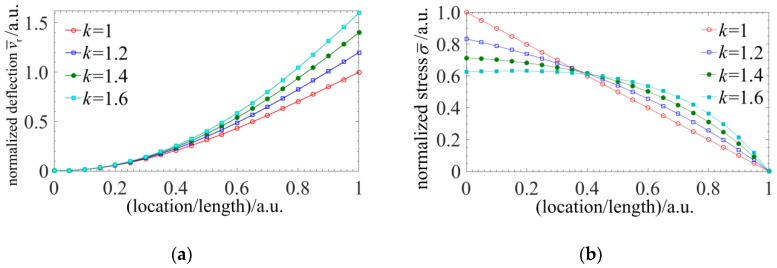
The normalized displacement and distributed stress curves of different taper ratios: (**a**) The normalized displacement curves; (**b**) The normalized distributed stress curves.

Through the analysis above, the normalized displacement and distributed stress curves of different taper ratios are calculated and illustrated in Figure 3. With the increase of the taper ratio, the deflection displacement of the spherical tip increases and the nonlinearity of the distributed stress within the stylus gets bigger. The most difference brought by the tapered four-cores FBG probe is the nonlinear distributed stress which would leads to the nonlinear chirp of the FBGs. However, there is no analytical expression between the spectrum shift and the nonlinear chirp FBGs, so a numerical solution should be utilized to achieve the clear relationship between the probe signal and the radial displacement.

### 2.2. Sensing Principle of the Axial Displacement

**Figure 4:**
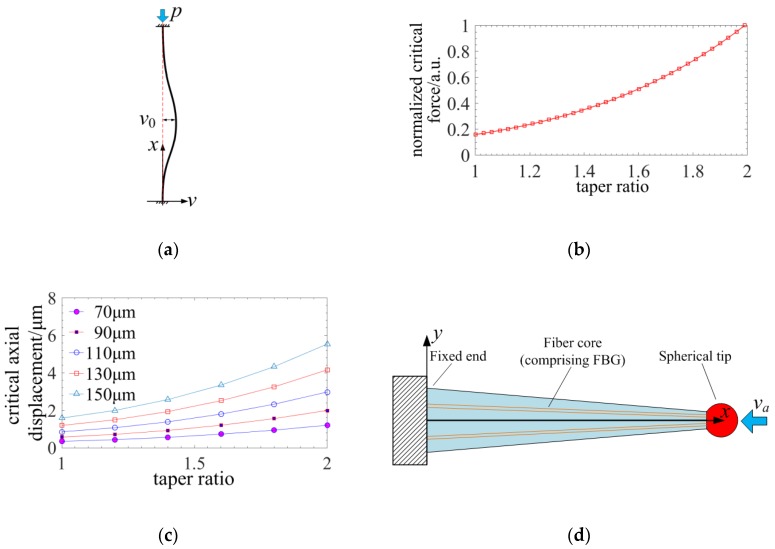
Analysis of the stability of a compressed tapered four-cores FBG probe: (**a**) Schematic diagram of the buckling probe; (**b**) The relationship between critical force causing deflection and taper ratio of probe stylus; (**c**) The relationship among critical axial displacement, diameter and taper; (**d**) Compressed beam model.

The tapered four-cores FBG probe is compressed under an axial tactile displacement. There are two phases of a compressed stylus. The first is stable compressing. Then, the stylus would be buckling when the axial tactile displacement exceeds a critical point. Figure 4a indicates the schematic diagram of the buckling probe.

During the axial sensing, the fixed end and the tactile end of the probe can be considered as a fixed support and a hinge support [17]. Based on the Sechler’s theory, the buckling curve can be expressed as:

(7) $$v_{B}(x)=v_{0}\left(1-\frac{x}{L}-\cos\frac{4.494x}{L}+\frac{1}{4.494}\sin\frac{4.494x}{L}\right)$$

wherein, $v_{0}$ is the maximum deflection displacement and L is the length of the stylus.

By applying the method of virtual work to a buckling stylus [17], the critical axial force causing buckling can be written as:

(8) $$P_{\mathrm{cr}}=\frac{\int_{0}^{L}EI(x){v_{B}{}^{\prime \prime }}^{2}(x)dx}{\int_{0}^{L}{v_{B}{}^{\prime }}^{2}(x)dx}=\frac{4.494\pi^{2}EI_{2}}{L^{2}}\left[\frac{k^{4}+k^{3}+k^{2}+k+1}{10}-\frac{3{\left(k-1\right)}^{4}}{64\pi^{4}}+\frac{{\left(k-1\right)}^{2}(k^{2}+k+1)}{8\pi^{2}}\right]$$

Figure 4b illustrates that the normalized critical axial force causing buckling increases with the taper ratio of the stylus. To build the relationship between the critical axial displacement and the structural parameters of the stylus, the axial displacement can be achieved through distributed strain as shown in Figure 4c.

(9) $$v_{a}=\int_{0}^{L}-\frac{4F_{a}}{E\pi {\left(D_{1}-\frac{D_{1}-D_{2}}{L}x\right)}^{2}}dx=-\frac{4LF_{a}}{E\pi kD_{2}^{2}}$$

wherein, $v_{a}$ is the axial displacement, $F_{a}$ is the axial force.

Substituting Equation (9) into Equation (8), the critical axial displacement can be re-written as:

(10) $$v_{a-\mathrm{cr}}=\frac{4.494\pi D_{2}^{2}}{16kL}\left[\frac{k^{4}+k^{3}+k^{2}+k+1}{10}-\frac{3{\left(k-1\right)}^{4}}{64\pi^{4}}+\frac{{\left(k-1\right)}^{2}(k^{2}+k+1)}{8\pi^{2}}\right]$$

Figure 4c shows that the critical axial displacement increases with the taper as a result of stiffness of the stylus and it can be 0.5–1 μm when the diameter of the stylus reaches 70 μm and the taper ratio approaches 2:1. It also indicates that a larger taper ratio would raise the stability of the axial measurement. A critical axial displacement of higher than 0.5 μm is enough for the axial sensing. When the axial tactile displacement is under the critical point, the compression of the tapered four-cores FBG probe is stable as shown in Figure 4d and the distributed stress can be expressed as:

(11) $$\sigma_{x}(x,y)=\frac{Ek}{L{\left(k-\frac{k-1}{L}x\right)}^{2}}v_{a}$$

**Figure 5:**
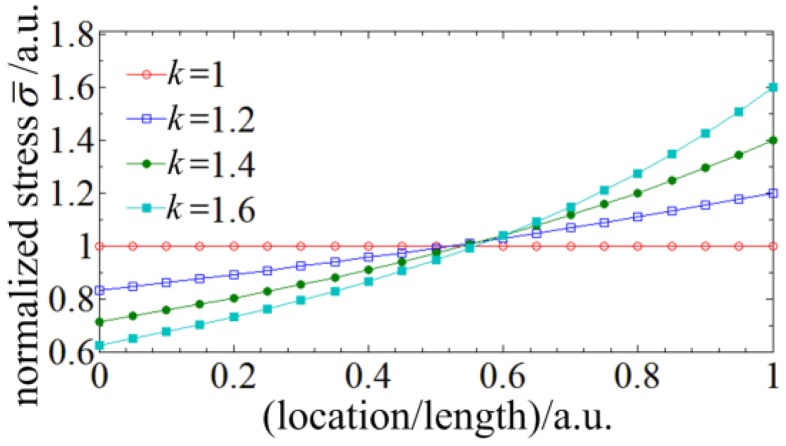
The normalized distributed stress curves of different taper ratio.

Figure 5 illustrates the distributed stress curves of different taper ratio. The nonlinearity of the distributed stress increases with the taper ratio and the nonlinear distributed stress leads to a non-analytical relationship between the spectrum shift and the axial displacement. So a numerical solution should be utilized to achieve the clear relationship between the probe signal and the axial displacement.

### 2.3. Sensing Characteristics of the Tapered Four-Cores FBG Probe

The analyses of the radial and axial sensing mechanism indicate that a nonlinear chirp is induced in the tapered four-cores FBG probe. To establish the relationship between the spectrum shift and the tactile displacement, a numerical solution proposed by Prabhugoud for solving the nonlinear chirp FBG’s spectrum is utilized to achieve the clear relationship between the probe signal and the tactile displacement [18].

#### 2.3.1. The Influence of the Taper Ratio on Sensing Accuracy

The tapered stylus would cause a nonlinear distributed stress which leads to asymmetrical spectrum distortion of the FBGs comprised in the probe. Simulations are run to investigate the influences on sensing accuracy. The parameters used in simulation are as follows: the lengths of the fiber taper and FBG are both 4 mm; the diameter *D*_1_ of the fixed end is 160 µm and the diameter *D*_2_ of the free end is 160 µm, 130 µm, 100 µm and 70 µm, respectively; the initial center wavelength of the FBGs comprised in the probe is 1550 nm; the distance between the FBGs and the neutral surface is a quarter of the stylus diameter; the effective refractive index *n_eff_* is 1.46; the amplitude of refractive index modulation *δn_eff_* is 2.5 × 10^−4^; the apodization function is in a Gaussian form.

**Figure 6:**
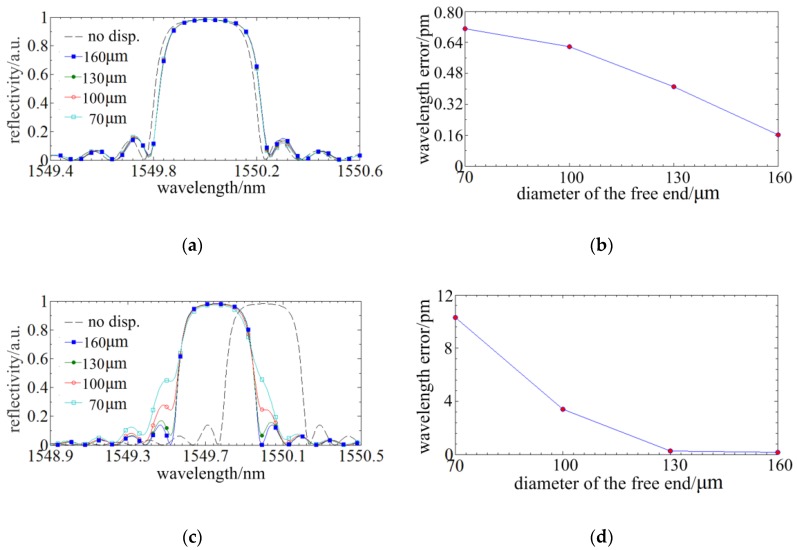
The influence of the taper on sensing accuracy: (**a**) The spectral responses of FBGs written in different tapers under a radial displacement of 10 µm; (**b**) The wavelength signal error of (**a**); (**c**) The spectral responses of FBGs written in different taper under an axial displacement of 1 µm; (**d**) The wavelength signal error of (**c**).

A radial displacement of 10 µm is loaded on the spherical tip of the probe and the spectral responses of FBGs written in different tapers are illustrated in Figure 6a. Based on the method in Ref [19], the sensing accuracy achieved by the phase correlation peak detection algorithm is the best, the centroid detection algorithm is worse and the maximum detection algorithm is the worst. The phase correlation peak detection algorithm is not suitable in this application because of the spectrum distortion [19]. So the centroid detection algorithm is utilized. The central wavelengths are calculated in different range of the spectrum reflection where the taper ratios of the probes and the displacements are different. The data of the central wavelengths verse the displacements is fitted into linear function. The maximum residual error or linearity is the wavelength signal error. Figure 6b illustrates the spectral responses and the wavelength error of the FBGs written in different tapers when the loaded displacement is 10 µm. It manifests that the wavelength error tended to increase as the taper ratio increases. The maximum error is ~0.72 pm.

Then, an axial displacement of 1 µm is loaded on the spherical tip of the probe and the spectral responses of FBGs written in optical tapers of different taper ratios are illustrated in Figure 6c. The distributed stress of a compressed stylus is much larger than that of a deflected stylus, so a greater the distortion or asymmetry is observed. Figure 6d indicates that the central wavelength error calculated by the centroid method could be ~11 pm when the taper ratio reaches maximum (when the diameter of the free end is 70 µm). Therefore, the taper induces a larger spectrum distortion under an axial displacement and has a higher influence on the axial sensing. It is necessary to propose a method suppressing the spectrum distortion of the nonlinear chirp of the FBG.

#### 2.3.2. The Improvement of the Phase-Shift Point on Spectrum Distortion

**Figure 7:**
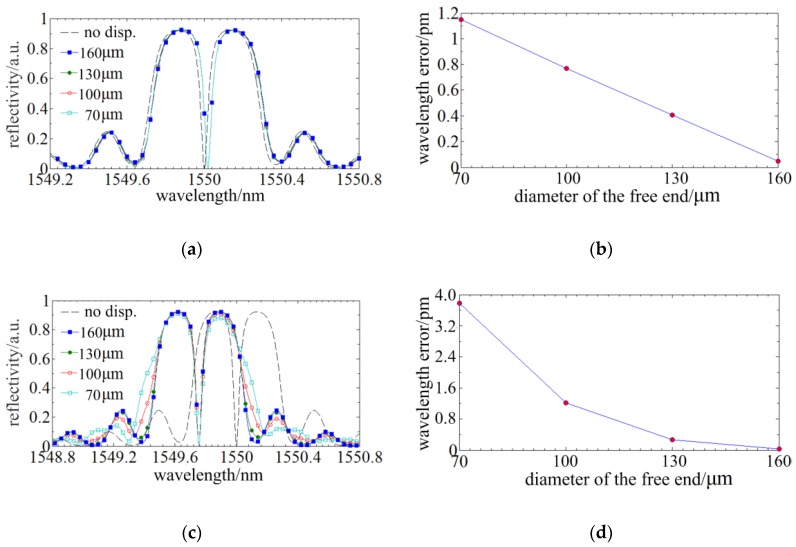
The improvement of the phase-shift point on the sensing accuracy: (**a**) The spectra of different tapers under a radial displacement of 10 µm; (**b**) The wavelength signal error of different tapers under a radial displacement of 10 µm; (**c**) The spectra of different tapers under an axial displacement of 1 µm; (**d**) The wavelength signal error of different tapers under an axial displacement of 1 µm.

In Section 2.3.1, the spectral distortion appears at the external profile and the nonlinear distributed stress has no influence on the internal spectrum structure. Through inducing a phase-shift point in the FBG, a fine structure would be generated in the spectrum. When the phase-shift is π/2 and the phase-shift point is located in the middle of the FBG, a fine spectrum structure of the highest depth and central symmetry could be achieved. Then, simulations, wherein the conditions are same as Section 2.3.1, are run to investigate the influences of the taper ratio on the distortion of the fine spectrum structure. Figure 7a,c demonstrate that the tapers have subtle influences on the fine spectrum structure induced by phase-shift point which indicates phase-shift point has benefit to suppress spectrum distortion. Figure 7b,d show that the wavelength signal error is downwards from 11 pm to 4 pm and the percentage of reduction is over 50%, so the phase-shift has a significant improvement of the sensing accuracy.

#### 2.3.3. The Sensing Characteristics of the Tapered Four-Cores Phase-Shift FBG Probe

The taper structure could reduce the scale of the tapered four-cores phase-shift FBG probe and extend the minimum measurable dimension. However, reducing the diameter of the fiber taper/fiber probe would cause it to lose its high sensitivity advantage [20]. To investigate the relationship between taper ratio and sensitivity, simulations of the probes of different tapers are run. The parameters are as follows. The length is 4 mm. The diameter *D*_1_ of the fixed end is 160 µm and the diameter *D*_2_ of the free end is 160 µm, 115 µm and 70 µm, respectively.

**Figure 8:**
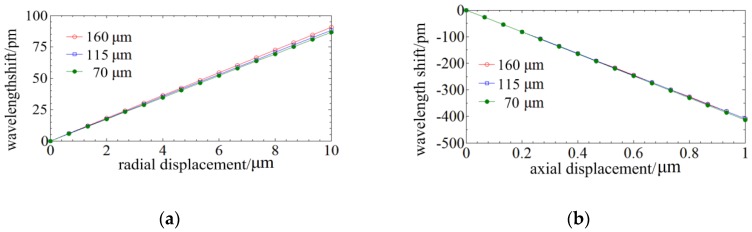
Signal curves of the phase-shift FBG probes of different tapers under displacements: (**a**) Signal curves of phase-shift FBG probes of different taper ratios under radial displacements; (**b**) Signal curves of phase-shift FBG probes of different taper ratio under axial displacements.

Figure 8 illustrates the signal curves of the probes under radial and axial displacements. It can be concluded that taper ratio has nearly no influence on the axial sensitivity and has a sensitivity reduction of less than 10% on the radial sensitivity. The sensitivity of the probe is proportion to the distance to the neutral plane. However, the sensitivity of the non-tapered four-cores FBG probe decreases ~60% when the diameter is reduced from 160 µm to 70 µm [20], so the tapered four-cores phase-shift FBG probe could improve the sensitivity when the diameter of the stylus is decreased.

**Figure 9:**
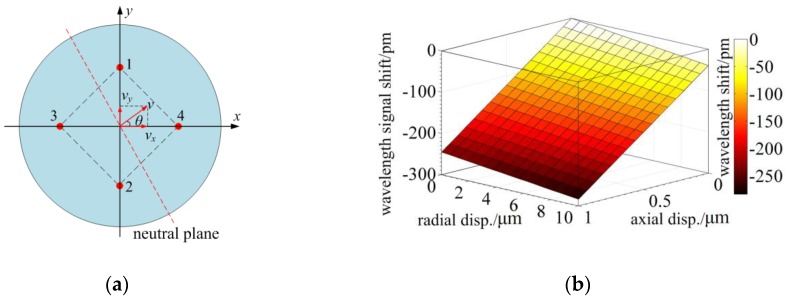
The three-dimensional sensing capacity: (**a**) The orthorhombic decomposition of the radial displacements; (**b**) The wavelength shifts verse the radial and axial displacements.

The tapered four-cores phase-shift FBG probe has three-dimensional sensing capacity. Based on the analysis of Section 2.1, the FBGs located in the neutral plane are not sensitive to the radial displacements. Therefore, there is a decoupling signal processing method when the four cores are in a square array as illustrated in Figure 9a. In this case, the radial displacement can be decomposed into two orthorhombic radial displacements along the diagonal line. These two orthorhombic displacements can be demodulated using the difference wavelength shifts of the FBGs located out of the neutral plane. The radial displacement can be expressed as:

(12) $$\left\{ \begin{array}{l} v_{x}=\left(\Delta \lambda_{3}-\Delta \lambda_{4}\right)/k_{x}\\ v_{y}=\left(\Delta \lambda_{2}-\Delta \lambda_{1}\right)/k_{x} \end{array}\right.$$

wherein, $\Delta \lambda_{i}$ is the wavelength shifts of the FBG*_i_* (*i* = 1,2,3 and 4), $k_{x}$ and $k_{y}$ are the sensitivity on axes *x* and *y*.

To investigate the coupling relationship between axial and radial displacements, the wavelength shifts of one FBG are simulated under both axial and radial displacements. The axial displacement is in a range from 0 µm to 1 µm and the radial displacement is in a range from 0 µm to 10 µm. Simulation result is demonstrated in Figure 9b and it indicates that the wavelength shift linearly varies verse axial and radial displacements. The fitting result is a plane equation. The regression coefficient R≈1 and relative residual error is less than 0.040%, so simulation results verify that the wavelength shift has a linear superposition of the axial and radial displacements. The axial displacement can be demodulated using an external temperature reference FBG:

(13) $$v_{z}=\left(\Delta \lambda_{1}+\Delta \lambda_{2}+\Delta \lambda_{3}+\Delta \lambda_{4}-4\Delta \lambda_{\mathrm{temp}}\right)/k_{z}$$

wherein, $\Delta \lambda_{\mathrm{temp}}$ is the wavelength shifts of the external temperature reference FBG, $k_{z}$ is the sensitivity on axis *z*.

Therefore, a three-dimensional displacement can be decoupled into three displacements into a Cartesian coordinate when the four cores are in a square array as illustrated in Figure 9a. By utilizing this method, a three-dimensional tactile sensing can be achieved with the tapered four-cores phase-shift FBG probe.

## 3. Experiments

In this section, experimental setups and experiments are demonstrated. First, the manufacturing method based on capillary self-assembly technique for the tapered four-cores phase-shift FBG probe are introduced in detailed. Then, a microcoordinate machine system is setup to do performance tests of the probe.

### 3.1. The Manufacture Method Based on Capillary Self-Assembly Technique for the Probe

**Figure 10:**
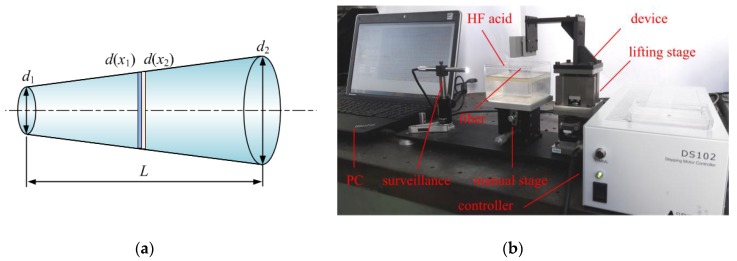
Manufacture of the tapered optical fiber: (**a**) Schematic diagram of the tapered optical fiber; (**b**) The etching device for the tapered fiber.

How to form the structure of the tapered four-cores phase-shift FBG probe is the key point of this micro-scale sensing method. Commercial available multicore fiber is an obvious choice, however, inscribing phase-shift FBG on the four-cores fiber, optical signals fanout and the design of structural parameters are hard to carry out. Especially, the taper stylus cannot be formed by the commercial multicore fiber. A method based on a capillary self-assembly technique has been proposed to manufacture the four-cores FBG probe and this method avoids the disadvantages above by using the commercial multicore fiber [10]. To form the taper stylus, the phase-shift FBG is pre-processed by HF acid etching. The decrease velocity of the fiber diameter is approximately constant. Figure 10a illustrates that the geometry structure of the stylus can be precisely controlled by setting etching time of each cross section and the difference of etching time can be derived as:

(14) $$\Delta t=\frac{d(x_{1})-d(x_{2})}{S}=\frac{\Delta d(x)}{S}$$

wherein, $d(x_{1})$ and $d(x_{2})$ are the diameter at position $x_{1}$ and $x_{2}$, S is the etching velocity.

To achieve a conical fiber, the etching time of each cross section is linearly varied and the velocity of the fiber entering HF acid is constant. During the experiments, a 60% concentration of HF acid is used and the etching velocity is ~30 µm/h. The structural parameters of the desired tapered fiber are as follows. The taper length is 5 mm, the diameter of the tip is 53 µm and the diameter of the other end is 125 µm. According to Equation (14), the etching processing time is 2.4 h and velocity of fiber entering HF acid is 0.278 μm/s. HF acid easily volatilizes and a layer of isooctane is used as isolation layer to avoid etching the fiber part outside the HF acid. Figure 10b shows the etching device for the tapered fiber. A lifting stage is controlled by PC to generate micro constant velocity downwards to several nm per second and a surveillance system monitors the contact status between optical fiber and HF acid. Table 1 shows the average experimental results of two groups of tapered fibers diameter (each group had four fibers) verse its location and it indicates that the etching processing has a good repeatability.

Then, four tapered fibers forms a fiber bundle in square array through the capillary self-assembly technique with an external micro hole. The rigidity of the tapered fiber is higher than that of the cylindrical fiber when their free-end diameters are same:

(15) $$F_{E}=\frac{3\sqrt{2}\pi Er^{4}}{4l^{3}}v$$

where, *E* is the Young’s modulus of the optical fiber, *r* is the diameter of the fiber, *l* is the length of the fiber, *v* is the deflection displacement of the fiber’s free end.

**Figure 11:**
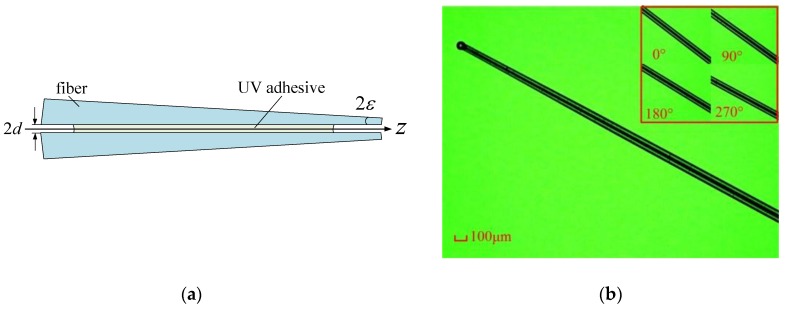
Manufacture of the tapered four-cores phase-shift FBG probe: (**a**) Schematic diagram of the self-assembly of the tapered optical fiber; (**b**) The photography of the tapered four-cores phase-shift FBG probe.

Therefore the critical length to form a square array for four cylindrical fibers is still effective for the tapered fibers [10]. On the other hand, the capillary bridge force between fibers brings the fiber getting close and contacted, so the self-assembly result of two tapered fibers is shown in Figure 11a and the generatrix lines are parallel. Finally, a tapered four-cores phase-shift FBG probe is manufactured through the capillary self-assembly fabrication process proposed in Ref [10]. Figure 11b shows the photography of the probe and its structural parameters measured by OGP MV200 from Quality Vision International Inc. (Rochester, NY, USA) are as following. The length is 3.2 mm, the diameter of the free end is 75.0 μm, the diameter of the free end is 105.2 μm and the taper ratio reaches ~1.4:1.

### 3.2. Experimental Setups for the Three-Dimensional Micro-Scale Sensing System

Experimental setups for the three-dimensional micro-scale sensing system consist of the experimental device and the microscale sensing system.

**Figure 12:**
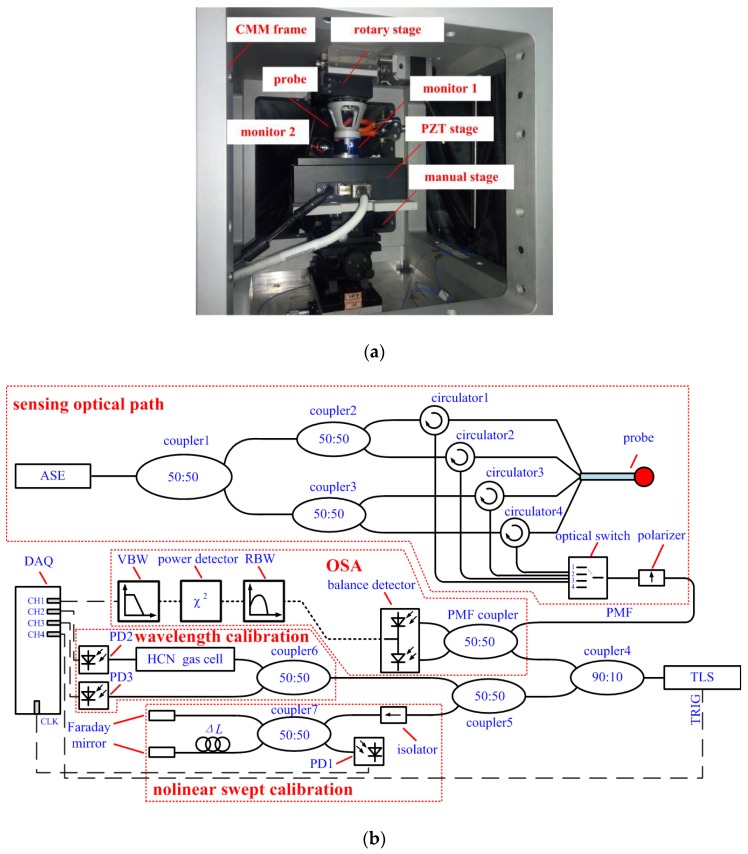
Experimental setups the three-dimensional micro-scale sensing system: (**a**) The photography of the experimental device; (**b**) Schematic diagram of the micro-scale sensing system.

Figure 12a illustrates the photography of the experimental device (a microcoordinate measurement machine, or micro CMM). The experimental device has a CMM frame for installing various kinds of modules, a rotary stage for aligning the probe with the coordinate of the CMM frame, a three-dimensional PZT stage for generating micro displacement (traveling range is 7 mm, resolution is 1 nm and repeatability is ±5 nm), a manual stage for tuning the position of the workpiece, two orthometric monitors for monitoring the relative position between the probe and the workpiece, and a probe for micro-scale sensing.

A microscale sensing system shown in Figure 12b is utilized to achieve the three-dimensional tactile displacements. The micro-scale sensing system consists of three parts, sensing optical path for making the probe working, OSA based on optoelectronic equivalent narrowband filtering for demodulating sensing signal [21], nonlinear swept calibration for equal wavelength interval sampling and wavelength calibration for improving absolute wavelength accuracy. The wavelength accuracy is determined by HCN gas cell from Wavelength Reference Company (Corvallis, OR, USA,) and it is ±0.2 pm. The resolution is 48 fm. To compensate the temperature shift of the FBGs written in the probe, a temperature compensation FBG is simultaneously sampled and the three-dimensional displacements are extracted by the method described in Section 2.3.3.

### 3.3. Experiments on Performance Tests of the Three-Dimensional Micro-Scale Sensing System

In this section, the proposed tapered four-cores phase-shift FBG probe is experimentally tested to demonstrate its sensing characteristics and three-dimensional sensing performance.

In Section 2.1, simulation results indicated that the phase-shift point is effective to improve axial sensing accuracy. This sensing characteristic is experimentally verified. A tapered four-cores FBG probe consisting of two common FBGs and two phase-shift FBGs is tested. The taper ratio is 2.04:1, the length is ~4.2 mm and the stylus diameter of the free end is 71.3 µm. During the experiments, spectra of both phase-shift FBG and common FBG are detected using the OSA in Figure 12b.

**Figure 13:**
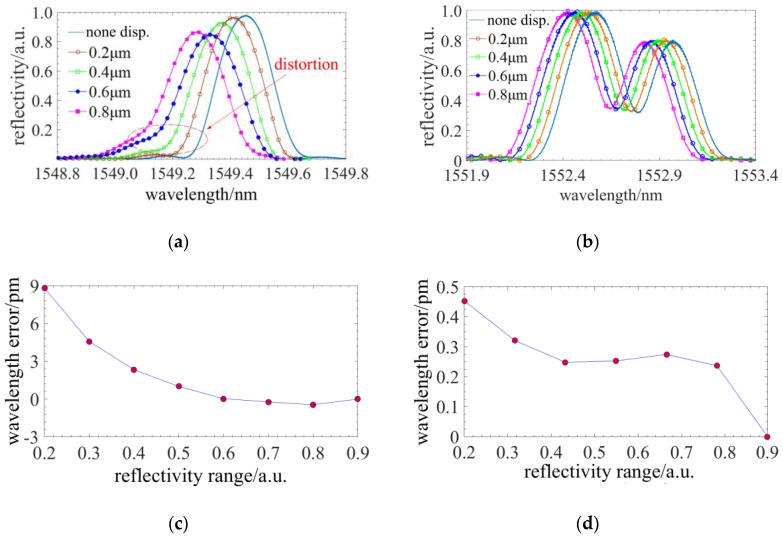
Experiments on the influences of the taper ratio on spectrum distortion: (**a**) The spectra of the FBG comprised in the probe; (**b**) The spectra of the phase-shift FBG comprised in the probes; (**c**) The wavelength signal error of (**a**); (**d**) The wavelength signal error of (**b**).

Experimental results are illustrated in Figure 13a,b. The spectrum of the common FBG appears distortions with the increase of the axial displacements. However, the forms of fine spectral notch structure of the phase-shift FBG remain stable. Then, the influence of spectral distortions on the axial sensing accuracy is investigated and the central wavelength of different reflectivity range versus displacements is derived to the central wavelength errors. Figure 13c,d show the maximum central wavelength error is reduced from 9 pm to 0.45 pm by directly detecting the fine spectral notch structure shift of the phase-shift FBG. The full range wavelength shift can reach ~160 pm and the nonlinearity is improved from 5.6% to 0.28% by using the phase-shift FBG. The simulated results in Figure 6b and Figure 7d indicate the maximum wavelength error of different taper ratios. However, Figure 13c,d illustrate the wavelength error of the taper ratio 2.04:1, so their representations are different and their results do not totally correspond to each other. On the other hand, the experimentally measured spectra are a little different from the simulations. The cause of this divorce, as far as the authors believe, is that the connection between the FBGs was not ideally rigid. When contacted by microparts, the ultraviolet glue inside the FBG probe could absorb part of the stress loaded on the probe. As a result of this, the practical nonlinearity and spectral distortion could be smaller than stimulated. Furthermore, experiments on sensing radial displacement were also conducted and the maximum wavelength error is nearly 0.2 pm, the same as the repeatability of the OSA.

Then, experiments were conducted to evaluate the influences of the taper on sensitivity. A non-tapered optical fiber and two tapered four-cores phase-shift FBG probes with different taper ratios of 1.52:1 and 2.04:1 are tested. The length of these FBG probes is ~4 mm and the diameter of their fixed end is 145.5 µm. The minimum diameter of their free end is 71.3 µm and the minimum diameter of the spherical tip is 99.24 μm. The maximum measurable ratio of depth to dimension is over 20:1.

**Figure 14:**
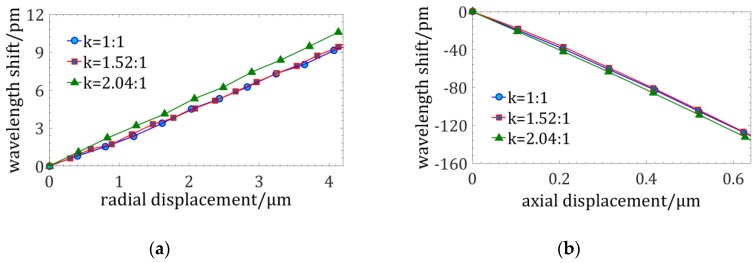
Experiments on the influences of the taper ratio on sensitivity: (**a**) The radial sensitivity curves; (**b**) The axial sensitivity curves.

Figure 14 shows the experimental results and it indicates that the radial and axial sensitivity nearly do not vary with the taper ratio. The maximum difference among the radial and axial sensitivity of different taper ratios are respectively 13% and 4%. The diversity may be caused by the precision of the stylus length which is manually controlled (its precision is lower than 0.5 mm). However, the radial sensitivity of a cylinder four-cores FBG probe is linearly decreasing with the diameter. The design of the tapered stylus can extend the minimum measurable dimension by twofold and has nearly no influence on its sensitivity.

Therefore, the sensing characteristics of the tapered four-cores FBG probe are experimentally verified. Experimental results demonstrate that the phase-shift point can effectively suppress spectrum distortion and the design of the tapered stylus can extend the minimum measurable dimension at lower cost of sensitivity. Next, experiments are conducted to test the performance of the probe.

**Figure 15:**
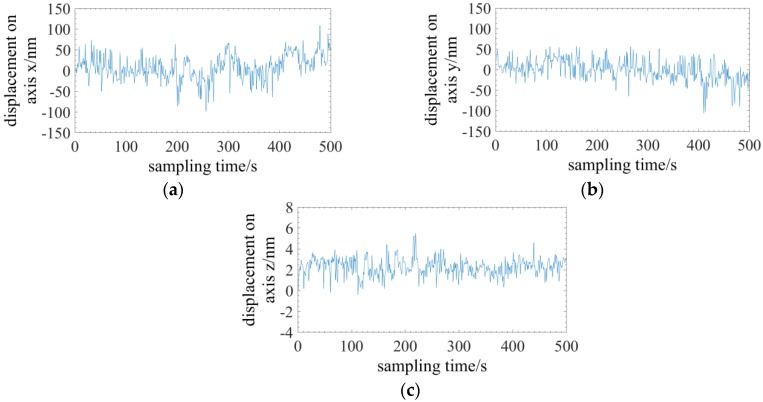
Experiments on the three-dimensional stability: (**a**) The stability on axis *x*; (**b**) The stability on axis *y*; (**c**) The stability on axis *z*.

The three-dimensional stability of the tapered four-cores phase-shift FBG probe is tested first. The taper of the probe is 2.04:1, the diameter of the fixed end is 105.5 µm and the length is ~4 mm. The three-dimensional displacements are achieved with the proposed method demonstrated by Equations (12) and (13). The measuring time of a single contact point is less than 20 s and the stability within an observation time of 500 s is illustrated in Figure 15. Experimental results indicate that the radial and axial stability is better than 200 nm and 10 nm, respectively.

**Figure 16:**
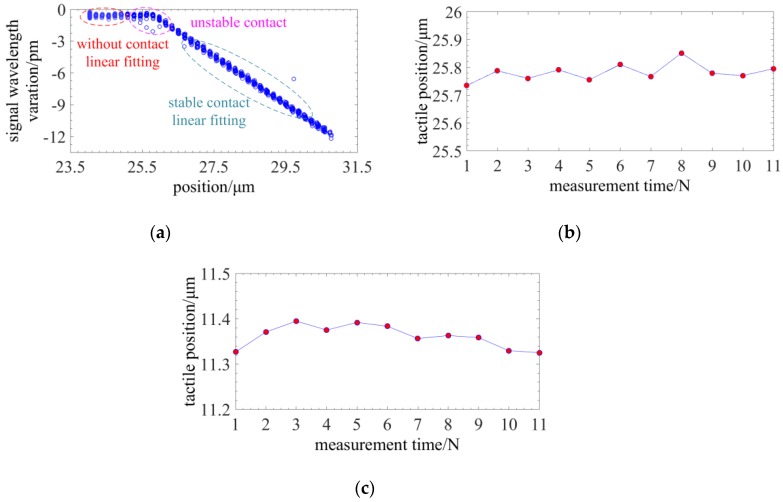
Experiments on the repeatability: (**a**) The tactile curve; (**b**) The repeatability on radial axis; (**c**) The repeatability on axial axis.

Then, the repeatability of the three-dimensional measurement is tested. Figure 16a shows the tactile curve and it indicates that the measurement consists of three phases [20]: (1) Without contact. The signal of the probe is not varying with the position of the probe. (2) Unstable contact. The signal of the probe is irregularly varying with the position of the probe as a result of weak interaction such as capillary force, electrostatic force and Van der Waals force. (3) Stable contact. The signal of the probe is linearly varying with the position of the probe. With the method proposed in the Ref [20], the signal curve in phase 1 is fitted to line A of zero slope and signal curve in phase 3 is fitted to line B. The crossing point between line A and B is the tactile point. The radial and axial tactile points are repeatedly achieved in 11 times. Figure 16b,c indicate that the repeatability of the three-dimensional measurement can be better than 31.1 nm.

**Figure 17:**
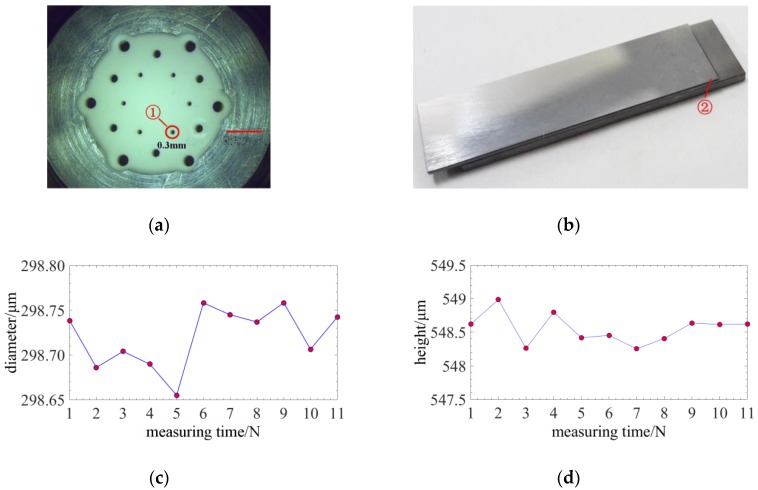
Experiments on measuring three-dimensional structures: (**a**) The micro and deep hole workpiece; (**b**) The micro step workpiece; (**c**) The measurement result of the micro and deep hole; (**d**) The measurement result of the micro step.

Finally, the three-dimensional structures of two workpieces are measured using this method. One of them, as shown in Figure 17a, is a micro and deep hole with a normal diameter of 300 µm. Its diameter at the depth of 1 mm is measured. The other is a micro step with a normal height of 500 µm as shown in Figure 17b. Experiments on measuring the diameter of the micro hole and the height of the step are to test the radial and axial measuring performance. Figure 17c,d illustrate the measurement results and the standard deviation (STD) representing the measurement precision is less than 150 nm. So this method can achieve high three-dimensional measurement precision. The measurement errors of the micro hole and micro step may be caused by cosine error (diameter bias or misalignment between axis z and the axis of the height) and the gap between two gauge block.

The repeatability for measuring single contact point is ~31.1 nm. The experimental setup for Figure 16b,c is measuring single contact point. The measuring work piece is a ring gauge and gauge block. However, Figure 17c,d experimentally tested the measuring results of actual work pieces. Two points on the work pieces are measured and the measuring results are calculated by their difference value. The accuracy of two measured points influences the final results. So they are worse than the repeatability for measuring single contact point and they are related to the precision of the measurement system.

## 4. Conclusions

In this paper, a three-dimensional micro-scale sensing system based on the tapered four-cores FBG probe is proposed. The radial and axial sensing models are built to investigate the microscale sensing mechanism. The design of the tapered stylus is found to induce a nonlinear distributed stress in the probe that would cause spectrum distortion. A π/2 phase-shift point is brought into the FBGs comprised in the probe to introduce a fine spectral notch in the center of the spectrum and it has an effective suppression on spectrum distortion. On the other hand, the tapered four-cores FBG probe has such sensing characteristics as extending the minimum measurable dimension at lower cost of sensitivity and three-dimensional displacement decoupling processing method. Then, a manufacture process based on capillary self-assembly is proposed to form the probe. Experiments are conducted to verify the sensing characteristics and micro-scale sensing performance. Experimental results indicate that the phase-shift point can reduce the central wavelength error of signal spectrum from 9 pm to 0.45 pm, which can improve the nonlinearity from 5.6% to 0.28% over the full measurable range. Furthermore, the design of the tapered stylus can extend the minimum measurable dimension by twofold and has nearly no influence on its sensitivity. The radial and axial stability is better than 200 nm and 10 nm, respectively. Moreover, the three-dimensional measurement repeatability can be better than 31.1 nm. Finally, a micro hole and a micro step are measured using the sensing system to evaluate its three-dimensional micro-scale sensing performance and three-dimensional measurement precision is 150 nm. The proposed three-dimensional micro-scale sensing system based on the tapered four-cores FBG probe could achieve a maximum measurable ratio of depth to dimension of higher than 20:1 and the minimum measurable dimension of ~100 µm. It has a broad application prospect in measuring micro-scale features for industry inspection or metrology.

## Figures and Tables

**Table 1:** Experimental results of the tapered fiber diameter verse its location.

Fiber Groups	5 mm	4 mm	3 mm	2 mm	1 mm	0 mm
#1	125.9 μm	113.5 μm	98.3 μm	83.3 μm	68.0 μm	53.5 μm
#2	125.7 μm	111.7 μm	97.6 μm	82.4 μm	66.1 μm	52.5 μm
Mean	125.8 μm	112.6 μm	98.0 μm	82.9 μm	67.1 μm	53.0 μm
